# Room-Temperature Visible-Light Single-Photon Avalanche Detection in Grating-Coupled Post-CMOS Photonic Circuits

**DOI:** 10.1021/acsphotonics.6c01473

**Published:** 2026-10-20

**Authors:** Marcus Westhues, Roman Burkard, Thomas Geruschke, Julia Hauser, Johannes Ewering, Stefan Dreiner, Aleksandar Nesic, Anna Lena Schall-Giesecke

**Affiliations:** † 28462 Fraunhofer Institute for Microelectronic Circuits and Systems (IMS), Finkenstr. 61, Duisburg 47057, Germany; ‡ Department of Electrical Engineering and Information Technology, 27170 University of Duisburg-Essen (UDE), Bismarckstr. 81, Duisburg 47057, Germany; § Center for Nanointegration Duisburg-Essen (CENIDE), Carl-Benz-Strasse 199, Duisburg 47057, Germany

**Keywords:** integrated optics, post-CMOS, waveguides, single photon detection, quantum photonics, SPAD

## Abstract

The integration of photonic circuits with on-chip single-photon detectors has emerged as a key enabling technology for highly scalable visible-wavelength photonic integrated circuits, underpinning advanced applications in quantum information processing and biophotonics. However, detecting weak optical signals down to the single-photon regime at specific wavelengths requires efficient interconnections between photonic waveguides and detectors. Here, we present an interconnect from silicon nitride photonic circuits to single-photon avalanche diodes (SPADs) on a standard 200 mm CMOS wafer integrated using post-CMOS integration. To realize this efficient interconnect, we optimized the interface by introducing metal reflector-assisted grating couplers for the target wavelengths 633 nm and 785 nm. To investigate the influence of the reflector on the efficiency of the interface, we conducted simulations and subsequent optimizations. In simulation, the grating-assisted interconnect achieves a coupling efficiency of 2.0 dB at 785 nm, representing a 6.4 dB improvement over the baseline design. To validate this improvement, we fabricated this structure with and without reflector and experimentally demonstrate detection of single photons at the target wavelengths. After post-CMOS integration, the utilized Fraunhofer IMS SPADs were operated at room temperature with an off-chip passive quenching circuit. Optical characterization showed an improvement by up to 4.5 dB by implementing reflectors. We show venues for improving the photon detection probability by correlating it with loss sources encountered in the fabricated devices. With these results, we demonstrate the applicability of post-CMOS integration and offer a photonic-integrated solution for detecting single photons from red to near-infrared wavelengths.

## Graphical abstract

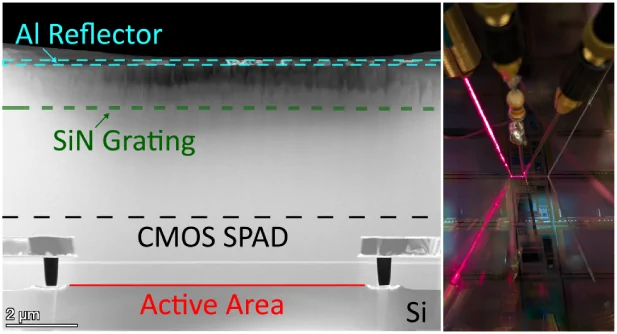

## Introduction

The scalable integration of single-photon detectors with photonic integrated circuits (PICs) is poised to unlock transformative capabilities across applications in quantum information processing^1–4^ and biosensing.^5–7^ As these applications advance from laboratory demonstrations toward practical implementations, the demand for chip-scale solutions that combine low-light detection with sophisticated photonic and electronic signal processing has become increasingly critical.^8,9^ Among the available single-photon detection technologies, single-photon avalanche diodes (SPADs) fabricated in complementary metal-oxide-semiconductor (CMOS) processes offer a compelling combination of room-temperature operation and scalability due to their compatibility with mature semiconductor manufacturing.^10,11^ When paired with silicon nitride (SiN) photonic platforms—which share similar CMOS-compatible processing—SPADs enable the realization of tightly integrated systems incorporating detectors, photonic routing elements, and application-specific integrated circuits (ASICs) on a single chip.

Despite this promise, a critical bottleneck remains: efficiently coupling light from SiN photonic waveguides to Si-based SPADs in a scalable, manufacturable fashion. The method of post-CMOS integration offers a wafer-level integration approach, where low-temperature processes are utilized to deposit and structure photonic layers directly on pre-fabricated CMOS wafers. Utilizing this method, the fundamental challenge lies in transitioning optical signals from horizontal propagation in the waveguide layer to vertical detection in the underlying SPAD active area, while maintaining high coupling efficiency, compact device footprint, and compatibility with monolithic integration schemes. A range of approaches has been explored, including evanescent coupling, angled metal mirrors, and grating couplers. While evanescent coupling offers broadband coupling with high coupling efficiencies, it requires the photonic waveguide and the Si active area of the SPAD to be brought into close proximity. This can either be achieved by defined Si mesas, which protrude from the Si substrate,^12,13^ or by introducing a gradual slope in the waveguide layer, which locally lowers the waveguide into proximity or contact with the SPAD.^14^ This requires either a monolithic process, in which SPAD and PIC fabrication is conducted in one continuous flow, or substantial process development and control to adiabatically redirect the waveguide’s propagation in the vertical dimension. Additionally, the SPAD’s dimensions need to be sufficiently large in the propagation direction to support efficient evanescent coupling, yielding increased dark count rates in the range of tens to hundreds of kilohertz.^12^ Coupling via an angled interface also poses a broadband solution to redirect light from propagation in the waveguide layer, but requires complicated etching processes to create the angled (metal) interface cutting through the waveguide structure.^15,16^ Grating couplers diffracting light toward the integrated SPAD can be a suitable solution, when no broadband operation is required. In that case they offer a compact geometry that allows for free placement and relatively simple fabrication atop the detectors. Because of the symmetry of the grating in the envisioned geometry, one half of the optical power is diffracted away from the detector. To redirect it back for detection, metal reflectors made, e.g., from aluminum (Al) may be implemented.^17–19^

In this work, we present the first demonstration of single-photon detection at visible-to-near-infrared wavelengths on a post-CMOS photonic integrated wafer, where the interface from waveguide to SPAD is realized by metal-reflector-assisted grating couplers. Two coupler geometries were optimized for 633 nm and 785 nm in the same silicon nitride photonic layer. To integrate a photonic layer with these structures onto Si-SPADs, the post-CMOS photonic platform at Fraunhofer IMS was used.^20^ The SPADs, which exhibit extremely low dark count rates of <1 Hz, were prefabricated at Fraunhofer IMS.^21,22^ After post-CMOS photonic integration, single-photon measurements were conducted in the system comprising SiN waveguides and the SPADs connected via the fabricated interface. We investigate the influence of the metal reflector on the coupling efficiency to the SPAD by analyzing the measured transmission in devices with and without a reflector. We estimated this transmission by two additional methods for further analysis. Our results underscore the viability of post-CMOS SiN photonics for scalable, room-temperature single-photon detection across the red and near-infrared spectra.

## Methods

### Device Design

The given geometry of the SPADs imposed constraints in designing the waveguide-SPAD interface, which is depicted schematically in Figure 1a. The locally doped Si substrate was covered by a 725 nm thick layer stack of isolators, e.g., thermally grown silicon dioxide (SiO_2_) and SiO_2_ deposited by plasma-enhanced chemical vapor deposition (PECVD). A structured layer of aluminum (Al) coated with titanium nitride (TiN) served as contact with a thickness of 550 nm. Above the active area of the SPAD, this metal layer was shaped as a ring with an inner radius of 5 μm and an outer radius of 6.5 μm. Through the gap in this ring, light needed to be guided into the SPAD. Lastly, the wafer was covered by a SiO_2_ passivation that was planarized to a target thickness of 650 nm above the contact metal layer. In this state, the wafers were handed over for post-CMOS photonic integration.

**Figure 1:**
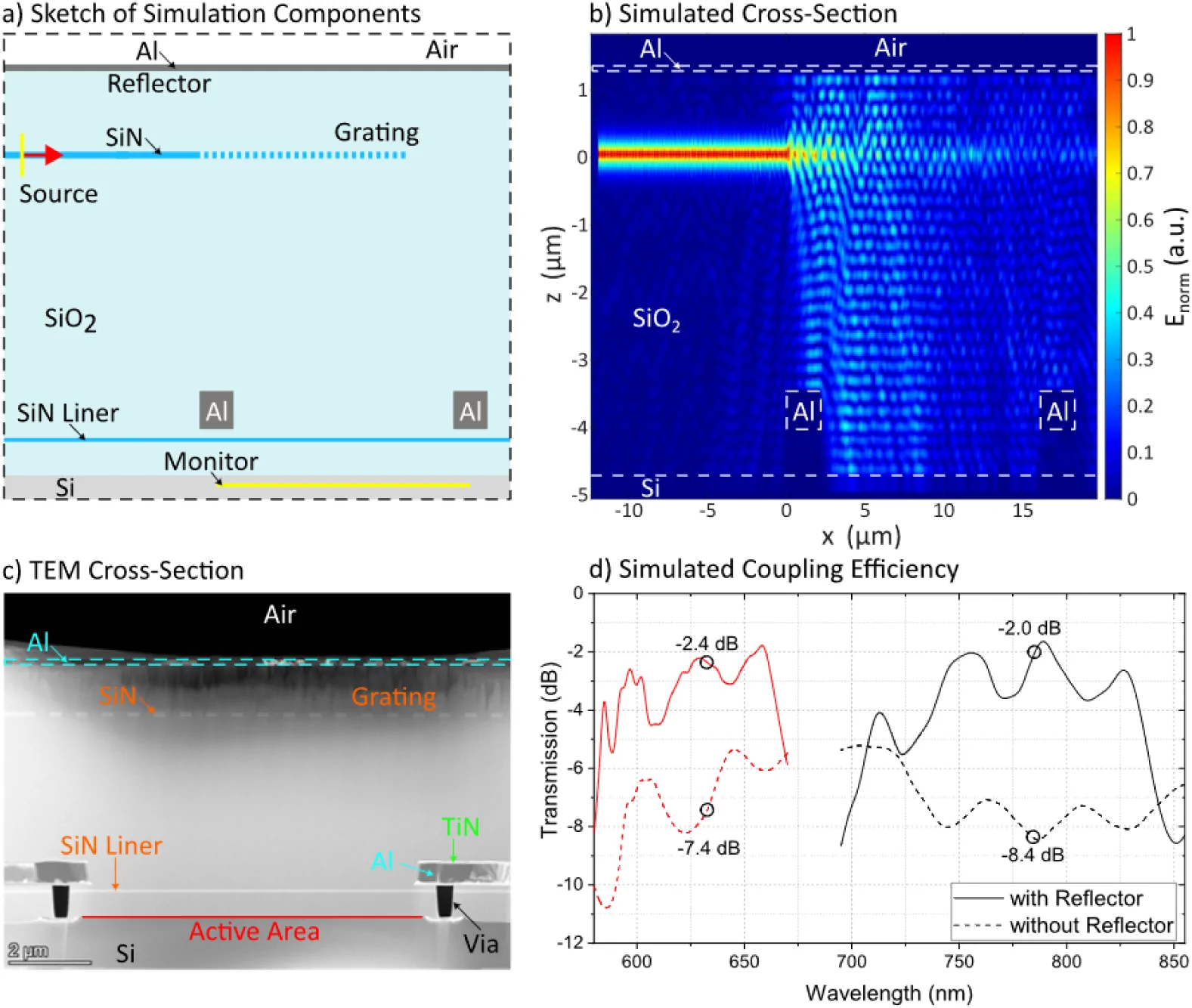
a) Sketch of the components featured in the simulations of the waveguide-SPAD interface. b) Simulated normalized electrical field for the wavelength of 633 nm from Lumerical FDTD. c) Annotated TEM cross-section of the fabricated interface. d) The comparison of optimal coupling efficiency of two separate structures, which were optimized for transmission with reflector at 633 nm and 785 nm, with the coupling efficiency in the same geometry but with removed reflector. Coupling efficiencies at the target wavelengths are marked.

Considering the SPAD geometries and the layer stack, 2D finite-difference time-domain (FDTD) simulations were performed to design the interface between the SPADs and the waveguides. A sketch of the simulated cross section is given in Figure 1b. The coupling interface was optimized regarding the transmission into the active area of the SPAD for the two target wavelengths, 633 nm and 785 nm. The interfaces for these wavelengths were to be fabricated on the same wafer, in the same photonic layer stack. Accordingly, the layer thicknesses for the SiO_2_ top and bottom cladding, as well as the SiN waveguide thickness, which were found in simulation constitute a compromise in transmission, where both wavelengths are near optimum. According to these simulations, the SiO_2_ thickness above the CMOS metal was increased to 3.45 μm. There, a 100 nm thick layer of silicon nitride (SiN) served as a waveguide layer. In it, single-mode waveguides, as simulated in Ansys Lumerical finite difference eigenmode (FDE) simulations, with a width of 620 nm and 1000 nm, were used for routing of light with λ = 633 nm and λ = 785 nm, respectively. This waveguide width was increased by adiabatic tapers to distribute the transmission across the active area of the SPAD. In further Ansys Lumerical 2D FDTD simulations, the optimal grating dimensions were determined. These required a 580 nm and 620 nm pitch for red and infrared light, respectively, with an equal fill factor of 50 %. In this geometry one half of optical power is diffracted away from the SPAD and lost into the air. To redirect this fraction back into the detector, a metal reflector was introduced. In further simulations, the layer thickness between waveguide and reflector, where interference of reflected light of both wavelengths from the metal’s surface with light diffracted toward the SPAD by the grating was constructive, was determined. This compromise, where both interfaces lie ∼0.5 dB below their possible maximum coupling efficiency, required a planarized cladding layer of 1.15 μm thick SiO_2_.

The optimized geometries were determined by a series of parameter sweeps monitoring the power transmission to the SPAD. The optical mode source was placed in the waveguide preceding the grating, and the power monitor spanning the extent of the physical SPAD was placed 100 nm beneath the Si surface. The monitored transmission only describes the power coupling from waveguide to Si SPAD; all effects pertaining the detection process in the active area and dissipation into the dead area of the 725 μm thick Si substrate are accounted for in the SPAD TCAD simulations.

This placement just below the Si surface was chosen for two reasons: First, to not additionally detect the reflected power fraction of light undergoing multiple reflections between Si SPAD and Al reflector and second to limit the influence of absorption according to Lambert-Beer’s law as light propagates through the Si substrate. These optimized geometries yielded transmissions of 58% (−2.4 dB) at λ = 633 nm and 63% (−2.0 dB) at λ = 785 nm. To investigate the influence of the reflector on the transmission spectrum, simulations were also carried out across a wavelength range. The simulated transmission spectra are given in Figure 1d and show the influence of the reflector on the coupling efficiency. At the target wavelengths of 633 nm and 785 nm, the addition of the reflector improves transmission by 5 dB and 6.4 dB, respectively. Due to the complex interference pattern caused by the layer stack found in the post-CMOS photonic integrated geometry, the coupling efficiency exhibits a considerable wavelength dependency. Nevertheless, the addition of the reflectors improves the coupling efficiency over the wavelength ranges from 580–670 nm and 710–840 nm.

It is important to note that this 2D simulation only approximates the 3D geometry of the real device. Divergence in the direction transverse to the simulated plane, as well as the ring shape of the contact metal through which light needs to pass to reach the SPAD, are examples for contributions that are not represented by this approximation. Thus, these simulations give an upper bound for the achievable coupling efficiency.

### Fabrication

The substrates for photonic integration were 200 mm wafers with SPADs, fabricated at Fraunhofer IMS in 0.35 μm high-voltage CMOS technology. The SPADs were structured via the LOCOS process as a planar CMOS junction and doped with boron. The active area of it has a circular shape with a diameter of 11 μm. It was covered with a layerstack of a total of 620 nm of SiO_2_ within which a 65 nm thick silicon nitride (SiN) liner was deposited via PECVD. This SiN liner is intrinsic to the underlying CMOS process. Connected to the anode and diode of the SPAD by vias, a contact metal of 550 nm Al was deposited and structured on top of these layers. Originally, the fabricated SPADs were designed for backside illumination. To enable the integration of a readout integrated circuit (ROIC), the SPAD wafer was encapsulated with a layer PECVD SiO_2_ that was planarized by chemical mechanical polishing (CMP) to a target thickness of 650 nm. Here, instead of the ROIC, the photonic circuit was integrated via a post-CMOS, wafer-scale manufacturing process. This process utilized optical DUV lithography for patterning. All values in the following are nominal unless specified differently. An initial layer of 2.8 μm SiO_2_ was deposited as a bottom cladding via plasma-enhanced chemical vapor deposition (PECVD). The waveguide layer of 100 nm SiN was deposited via a PECVD process tailored for transmission in the visible wavelength range^20^ and subsequently structured via reactive ion etching (RIE). Afterwards, a top cladding of 1.7 μm SiO_2_ was deposited, thinned, and planarized to 1.15 μm by CMP. This yielded a flat surface, which typically results in a SiO_2_ root mean square roughness of 0.3 nm in our processing, upon which a 100 nm aluminum (Al) reflector layer was deposited by physical vapor deposition (PVD) and subsequently patterned. The metal was encapsulated by a 200 nm SiO_2_ layer, before the Al pads of the SPAD wafer were opened by locally etching through 4.8 μm of SiO_2_ with RIE. A side view of the interface, captured by transmission electron microscopy (TEM) is shown in Figure 1c. A top-down view of the device as a schematic and a microscope image is given in Figure 2a and b.

**Figure 2:**
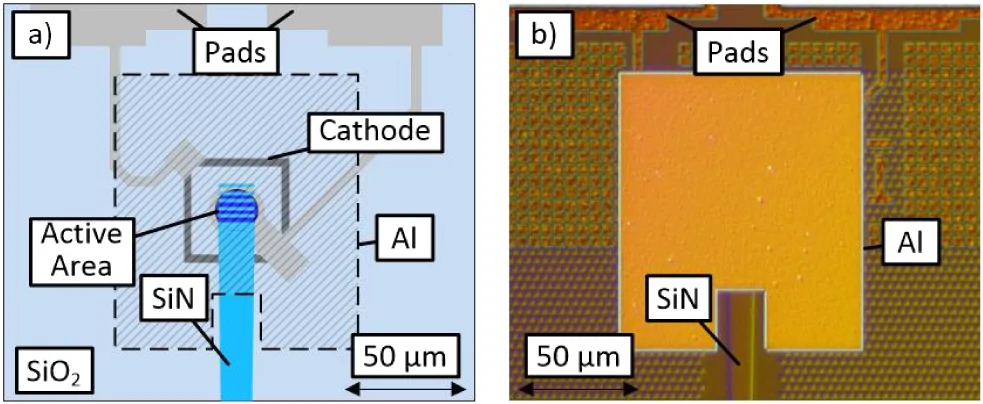
Top-down view of the waveguide-to-SPAD coupler as a) a sketch and b) a microscope image after fabrication.

### Characterization

For electrical characterization of the SPAD, the pads for the anode and cathode of the SPAD were contacted with tungsten probe needles. A passive quenching circuit was realized utilizing a 470 kΩ resistor in series as shown in Figure 3c. The bias voltage was applied via a Keithley 2614B source-measure unit, and the signal was measured using an Agilent Infiniium MSO9404A oscilloscope.

**Figure 3:**
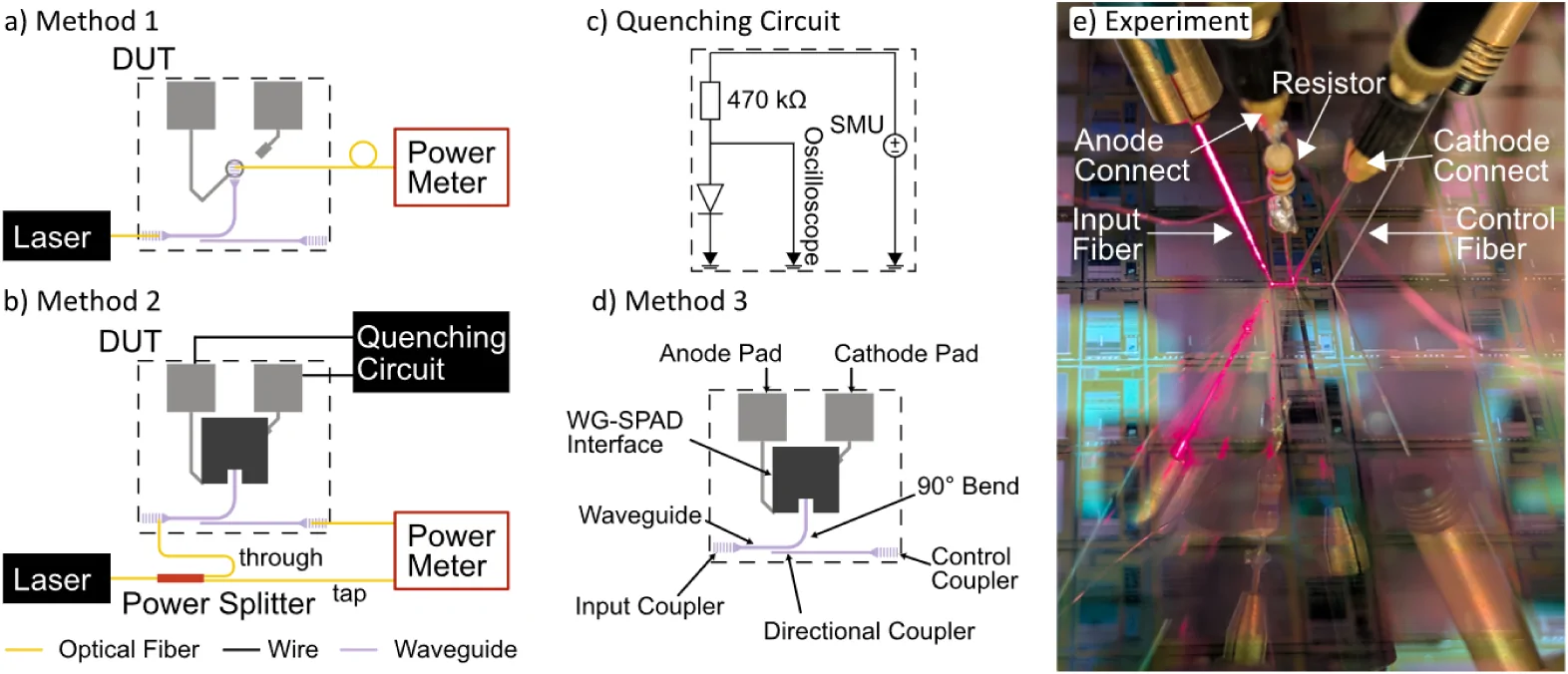
a) Method 1: Optical characterization with a fiber above the SPAD coupler, only applicable on devices without reflector. b) Method 2: SPAD measurement setup, conducted on devices with and without reflector. c) Passive quenching circuit implemented in SPAD measurements (Method 2). d) Method 3: Components of the optical path from input fiber to SPAD detection, as considered in the calculation of estimated transmission *T*_SPAD,estimated_ for all geometries. e) Picture of electrically and optically contacted SPAD in experiment.

To characterize the developed interface optically, a supercontinuum pulsed laser source (NKT SuperK FIANIUM FIU-15) emitting from 400–2400 nm was used, which was connected to a tunable bandpass filter with a full width at half maximum line width of 1.5 nm (up to a wavelength of 650 nm) and 2.5 nm for longer wavelengths. By controlling the coupling from the filter to the optical fiber, the output laser power was additionally attenuated. To set the single-photon condition, i.e., an average of <1 photon/pulse input to the device under test (DUT), a fiber-based optical power splitter with a 90:10 splitting ratio was used. By routing 90% of the laser output power to an optical power meter (OPM), the mean number of photons per pulse impinging on the DUT was monitored. With the laser repetition rate of 78 MHz and for the investigated wavelengths, the laser output was attenuated to a power reading at the OPM in the order of −70 dBm. The remaining 10 % from the FPS was guided to the DUT through a single-mode fiber (SMF). It was coupled into the photonic waveguides via an input grating coupler. An on-chip directional coupler split this input to two output grating couplers. The first, to which only a fraction of the optical power was tapped, was used to monitor the alignment of the input fiber. At this output, the emitted light was collected by a multimode fiber (MMF) and measured using an OPM. The second grating coupler, to which the main transmitted power fraction of the directional coupler was routed, connected the photonic waveguides to the SPAD, as previously explained in Figure 1. Two separate designs of this photonic-SPAD interface were designed, which was fabricated on separate dies, one for the target wavelength of 633 nm and one for 785 nm, respectively.

The SPAD was biased in Geiger mode and its output voltage was recorded using an oscilloscope. Photon detection events were defined as voltage pulses exceeding a fixed voltage threshold, and the number of such pulses was counted for each recorded waveform. To determine the average count rate of the SPAD *f*_SPAD_ (λ) as a function of wavelength λ, measurements were performed under illumination with total acquisition times of at least 4 s. For dark count rate measurements, acquisition times of up to 40 s were used to improve statistical accuracy.

For a pulsed laser source, the number of photons in an emitted pulse follows a Poisson distribution. The mean value is given by

$$\mathrm{n̅}=\frac{P_{\mathrm{avg}}\lambda }{f_{\mathrm{rep}}hc}$$

with the average optical power *P*_avg_ detected at the reference OPM, the repetition rate of the laser *f*_rep_, Plank’s constant *h* and the vacuum speed of light *c*. In the experiment, the laser output was attenuated until the laser pulses at the fiber output toward the DUT had an average number of photons *n̅*<1 to ensure that avalanches are only induced by single photons. The expected incident rate of photons at the DUT was determined via

$$f_{\exp}\left(\lambda \right)=\frac{\rho_{\mathrm{FPS},\mathrm{thru}}\left(\lambda \right)}{\rho_{\mathrm{FPS},\mathrm{tap}}\left(\lambda \right)}\cdot f_{\mathrm{rep}}\cdot \mathrm{n̅}$$

whereby ρ_FPS,thru_(λ) describes the power fraction split to the DUT by the fiber power splitter (FPS) and ρ_FPS,tap_(λ) denotes the power fraction guided to the OPM.

By comparing the measured count rate *f*_SPAD_(*λ*), after subtraction of the dark count rate *f*_DCR_, with the expected input rate of photons *f*_exp_(*λ*), the transmission to the integrated SPAD was determined using

$$T_{\mathrm{SPAD},\mathrm{meas}}\left(\lambda \right)\left[\mathrm{dB}\right]=10\cdot \log_{10}\left(\frac{f_{\mathrm{SPAD}}\left(\lambda \right)-f_{\mathrm{DCR}}}{f_{\exp}\left(\lambda \right)}\right)$$

The setup used in this method is depicted schematically in Figure 3b and an image of the contacted DUT in the experiment is given in Figure 3e.

It must be considered that the measured transmission T_SPAD,meas_ also includes contributions from other loss mechanisms, as visualized in Figure 3d. Namely, these contributions are the fiber-to-chip coupling loss η_CL,in_, the waveguide loss *a_WG_*, the loss from a singular 90° bend η_bend_, the bar transmission of a directional coupler η_DC,bar_ and the internal detection probability of the integrated SPAD η_IDP_. Therefore, the estimated SPAD transmission *T*_SPAD,estimated_ can inversely be determined by

$$T_{\mathrm{SPAD},\mathrm{estimated}}=\eta_{\mathrm{CL},\mathrm{in}}+a_{\mathrm{WG}}+\eta_{\mathrm{bend}}+\eta_{\mathrm{DC},\mathrm{bar}}+\eta_{\mathrm{CL},\mathrm{SPAD}}+\eta_{\mathrm{IDP}}$$

To determine these additional losses independently, dedicated test structures were added, where applicable. On the chips, where measurements on the integrated SPAD were taken, waveguide attenuation α—which by multiplication with the waveguide length L yields the waveguide loss *a*_WG_—and input coupling loss η_CL,in_ were determined by measurements of cutback structures and straight waveguides connecting two grating couplers. To extract the mean bend losses η_bend_, representative measurements of structures with a varying number of 90° bends were taken. To characterize the bar transmission of the directional couplers η_DC,bar_, which were previously designed utilizing Lumerical 3D FDTD simulations, transmission measurements were taken on structures containing one directional coupler. The normalized bar and cross transmission η_DC,bar_ and η_DC,cross_ were calculated from the measured spectra in the two combinations for bar transmission *T*_bar,1_ and *T*_bar,2_ and the two combinations for cross transmission *T*_cross,1_ and *T*_cross,2_ according to Chen et al.^23^ (see also for a figure).

Because no suitable test structures for characterization of waveguide-to-SPAD coupling were available, the coupling loss η_CL,SPAD_ was simulated in FDTD simulations. These simulations were done individually for each die, implementing process variations found from in-line measurements, e.g., from ellipsometry of layer thicknesses. The validity of such simulations was previously confirmed in an inverse geometry with a metal reflector below a grating coupler that coupled light to a fiber.

Lastly, the internal detection probability η_IDP_ of the integrated SPADs could not be characterized on-chip as no suitable test structures were available after post-CMOS integration. Thus, TCAD simulations were employed to predict η*_IDP_* by modeling the detection process, including photon absorption profiles, carrier generation and transport, and avalanche multiplication triggered by impact ionization. The simulations incorporated the device geometry and doping profiles as fabricated.

On selected dies for each wavelength, the aluminum reflector above the SPAD was removed. This allowed an additional independent measurement of the optical transmission in Method 1. In this configuration, which is schematically given in Figure 3a, a multimode fiber (MMF) was positioned above the SPAD site, and the transmitted optical power was recorded using an external power meter. The resulting optical transmission *T*_opt,meas_(λ) provides an estimate of the transmission through the photonic circuit and includes the contributions of the input fiber-to-chip coupling η_CL,in_, waveguide propagation loss *a*_WG_, the coupling coefficient of the direction coupler η_DC,bar_, and the losses introduced by the 90° bend η_bend_ This measurement additionally contains a contribution from chip-to-fiber coupling at the SPAD site. To compare it to the other measurement methods, this contribution is corrected by the difference in simulations of chip-to-fiber coupling and SPAD coupling with die-specific layer thickness information. After also correcting for the internal detection probability η_IDP_ of the integrated SPADs, this method is suitable for comparison with the SPAD-based transmission measurements.

## Results

After post-CMOS photonic integration, the SPADs were electrically characterized first. The I–V curves under reverse and forward bias were measured. Figure 4a depicts the result from an exemplary SPAD. It exhibits the expected SPAD characteristics, indicating that its core behavior remains consistent with that of a standard SPAD even after post-CMOS photonic integration. From the I–V curve, the breakdown voltage *U*_BD_ was extracted. The extracted *U*_BD_ of (33.68 ± 0.46) V, measured across 18 dies, agrees with the nominal value for this SPAD device. Dark count rate measurements yielded values as low as 0.002 cps/μm^2^, which lies at the low end of the technology in its non-integrated form. This indicates that integration-induced damage to the SPAD was minimal. Because no suitable test structures were available to quantify the SPAD’s internal detection probability η_IDP_, experimental conditions were chosen to resemble the simulated parameters. Specifically, an excess voltage *U*_exc_ of 5 V was set. With this excess voltage applied, the detection probability at the target wavelengths of 633 nm and 785 nm was found in TCAD simulations as 22.6% and 18.7%, respectively. The time trace of an avalanche current pulse is shown in Figure 4b. It exhibits a sharp rise due to the onset of avalanche breakdown, followed by quenching of the avalanche process by the passive quenching circuit, resulting in a full width half maximum time *t*_FWHM_ of 5.1 μs. This pulse duration does not represent the intrinsic response time of the SPAD, but rather results from the capacitances and resistances of the components in the external quenching circuit that was utilized.

**Figure 4:**
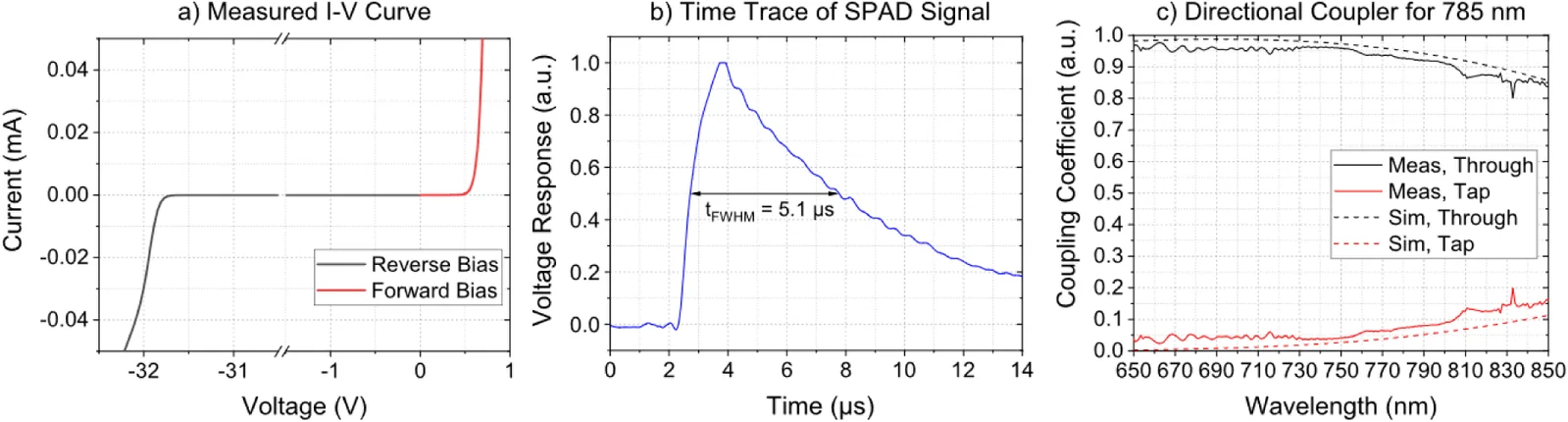
a) I–V curve of an exemplary SPAD under reverse and forward bias after post-CMOS photonic integration. b) Waveform of a pulse in the SPAD output voltage counted as a photon detection event. c) Comparison of the simulated and measured normalized coupling coefficients for a 95:5 splitter designed for 785 nm.

Of the photonic components connecting the input fiber to the SPAD, characterization is shown by example of the 95:5 directional couplers. Figure 4c depicts the measurement results on the device for 785 nm. The bar transmission of the directional coupler η_DC,bar_ was extracted from measurement of a single directional coupler as outlined in the methods section. At 633 nm, measurements yielded a bar transmission of 93.0%, as compared to a simulated 95.0%. At the wavelength of 785 nm, it was derived from transmission measurements as 92.5%, while simulations predicted 94.8%. Values from simulation and measurement align closely across the evaluated spectrum, with residual mismatch due to fabrication variations in, e.g., gap width.

By the methods outlined previously, the estimated transmission to SPAD was determined. The corresponding measurements were conducted on a set of eight dies per wavelength across two wafers, of which two dies contained the interface without reflector. Measurement results at the two target wavelengths are given in Figure 5.

**Figure 5:**
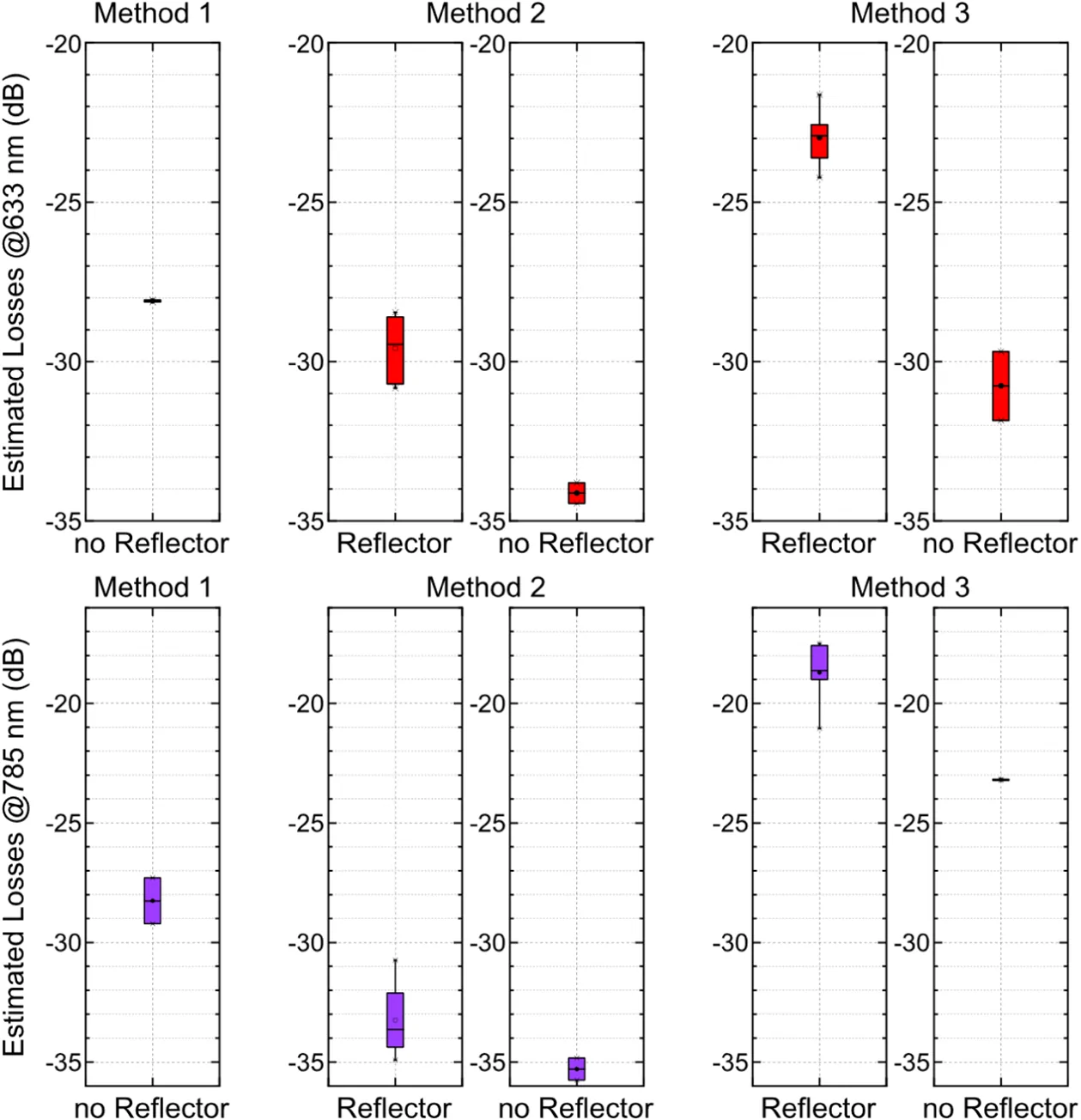
Three measurement methods are compared for a wavelength of 633 nm (top) and a wavelength of 785 nm (bottom), as well as the difference in transmission with and without reflector. In Method 1, the optical transmission to a fiber placed above a SPAD without a reflector is measured. In Method 2, the transmission is derived from the count rate measured at the integrated SPAD. For Method 3, transmission is calculated from the contributions of components found in the integrated system, which were determined by means of further test structures or simulations. Figure 3a, b, and d contains depictions of these three methods.

To evaluate the measurements and to flatten deviations from wafer-level process variations, the mean transmission across the various cases is considered. In SPAD measurements (Method 2) of the optimized case with a reflector, a signal with an average transmission of −29.6 dB is detected at 633 nm, and an average transmission of −33.2 dB is detected at 785 nm. Without the reflector, it drops to −34.1 dB and −35.3 dB at 633 nm and 785 nm, respectively.

These evaluated transmission values are compared to component-wise calculations of the estimated SPAD transmission *T*_SPAD,estimated_ (Method 3). For red light (633 nm), the individual contributors in the case with the top reflector on average amount to −11.9 dB from input coupling loss η_CL,in_ and −2.1 dB from waveguide loss *a*_WG_, respectively, found by means of cutback measurements, −0.3 dB from directional coupler bar transmission η_DC,bar_, −0.1 dB from a single 90° bend η_bend_, −2.3 dB from waveguide-to-SPAD coupling η_CL,SPAD_, and −6.5 dB from the SPAD’s IDP η_ICP_. For infrared light (785 nm) and in the case with the reflector, these contributions are on average: −7.2 dB from η_CL,in_, −0.6 dB from *a*_WG_, −0.4 dB from η_DC,bar_, −0.1 dB from η_bend_, −3.1 dB from η_CL,SPAD_, and −7.3 dB from η_IDp_. Similarly, component losses for the case without the top reflector were evaluated, but are not explicitly given here. The high contributions from waveguide loss are due to high attenuation on these fabricated wafers. At 633 nm, attenuation α as extracted by cutback measurements has an average of −9.9 dB/cm, while at 785 nm it is −3.3 dB/cm. These values exceed the previously achieved attenuation in the same platform with similar processing greatly.^20^ The source of this discrepancy is found in the processing of these experimental wafers, where even multiple cleaning steps could not fully remove photoresist residues on the structured waveguides, which is typically not encountered.

Across the variations with and without reflectors, as well as the two target wavelengths, estimation utilizing the added component transmissions (Method 3) exceeds the one from Method 2. In the configurations with reflector, the mean value of the added component transmission exceeds the SPAD transmission by 6.6 dB at λ = 633 nm and by 14.2 dB at λ = 785 nm. In the case without the reflector, deviations of 3.4 dB for red light (633 nm) and 12.1 dB for near-infrared light (785 nm) are determined. Thus, the calculations in Method 3 underestimate the losses measured at the SPAD in Method 2.

Sources for this offset are identified in the components, which cannot be characterized on-chip, namely the waveguide-to-SPAD coupling efficiency η_CL,SPAD_ and the detection probability of the SPAD η_IDP_. Besides possible deviations of layer thicknesses impacting the coupling efficiency, the reflectivity of the two metal interfaces with which the diffracted light interacts are sources for additional losses. One interface is found at the top reflector, while the other is found at the CMOS metal structure from which the first order of diffracted light was reflected. In simulations, this CMOS metal layer was implemented as a block of Al, but on the wafer, it was lined with a titanium nitride (TiN) coating. It can be seen in the TEM cross-section in Figure 1c and in Figure 2b as an orange coating on unopened metal pads, while the underlying white Al was revealed through pad opening. This layer’s anti-reflective properties are reduced in the red wavelength range, thus yielding a smaller offset from calculations at 633 nm in comparison to 785 nm.^24^

The transmission measured in Method 1 on devices without a top reflector supports this thesis, when comparing it to the estimated transmission from individual components (Method 3). Because in both Methods 1 and 3, the internal detection probability η_IDP_ is added as a fixed value, the relative difference between these two methods eliminates its contribution. For red light (633 nm) a lower transmission by −2.7 dB was calculated than optically measured, while for near-infrared light (785 nm) the calculated transmission exceeded the optical transmission from Method 1 by +5.1 dB.

The measurements further show the positive impact of the metal reflector. By introducing it, the measured mean transmission to the SPAD (Method 2) improved by 4.5 dB at λ= 633 nm and by 2.1 dB at λ= 785 nm. This measured improvement is lower than the one expected from component calculations (Method 3) at 7.8 dB and 4.5 dB, respectively. A non-unity reflectivity of the top Al reflector, influenced by material reflectivity and surface roughness, is a possible source of this mismatch.

From these measurements, an exact value for the coupling efficiency from waveguide to SPAD η_CL,SPAD_ cannot be extracted individually. Instead, an estimate for the upper boundary of this contribution can be made. For this estimate, the assumption is made that the difference in calculated transmission (Method 3) and the transmission measured at the SPAD (Method 2) is completely due to a decreased coupling efficiency. Thus, this difference is added to the simulated coupling efficiency η_CL,SPAD_ for the individual dies. In the case with a metal reflector, this estimates the coupling efficiency as >−9.5 dB at 633 nm and >−17.9 dB at 785 nm. Without a reflector, this approach yields >−12.5 dB at 633 nm and >−19.9 dB at 785 nm. It is important to note that this estimate considers the internal detection probability of the SPAD η_IDP_ to match the simulation perfectly, which is not verified on these wafers. Also, the discrepancy between Method 3 and the optical transmission measurement of Method 1 indicates that the calculated losses do not describe the system perfectly, which indicates a better coupling efficiency than given by this estimate.

On dies without a top reflector, measurements were taken on a 30 mm wide spectrum surrounding the target wavelength. These measurements were conducted by setting up the experiment for the target wavelength and successively adjusting the wavelength without changing further experimental parameters, e.g., input fiber coupling angle or output laser power. The evaluated transmission single, exemplary dies for each wavelength via the three methods are shown in Figure 6.

**Figure 6:**
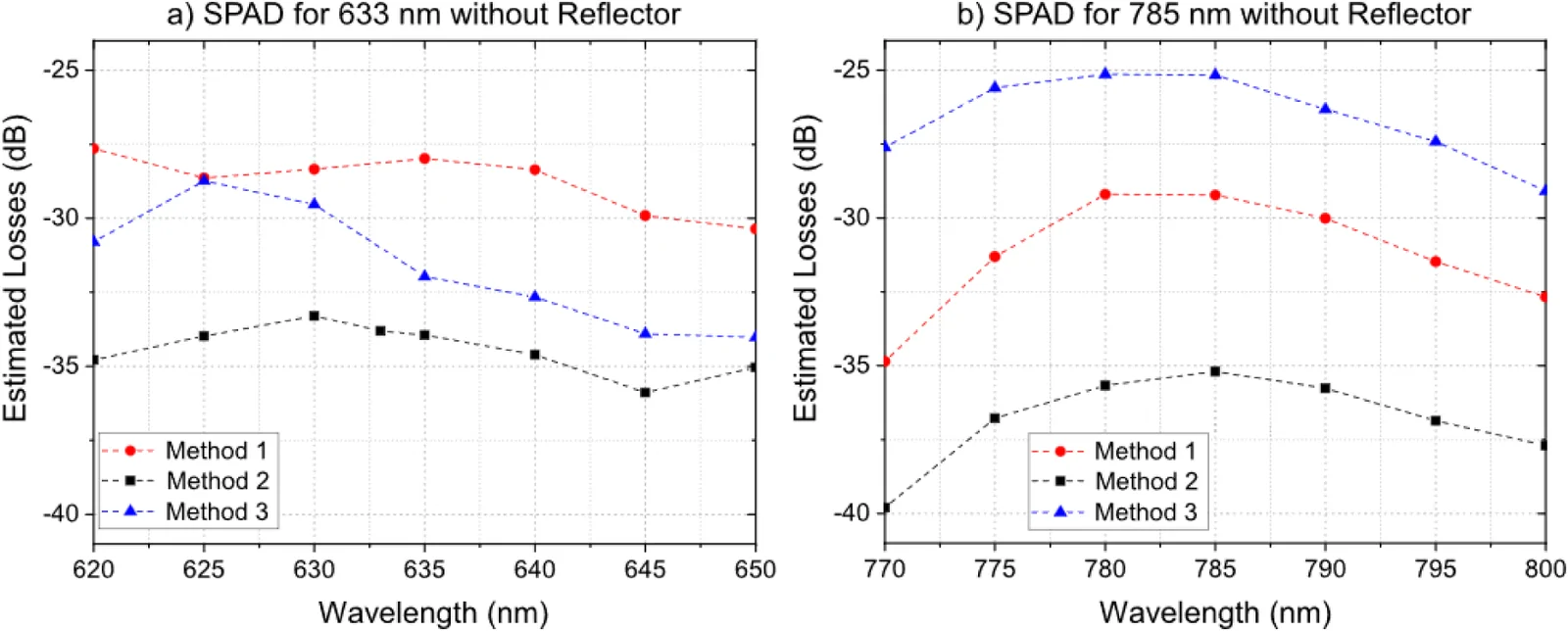
Spectral distribution of transmission in a 30 nm section surrounding target wavelengths. Measurements with the three methods were conducted on the same structures for the respective wavelengths.

Qualitatively, the behavior of the three evaluated spectra is in good agreement. In the near-infrared they exhibit a peak transmission at 780–785 nm, from which the transmission gradually decreases. At red wavelengths, no clear maximum is visible, but the transmission rather oscillates within a band. Positions of slopes within these variations differ between the measurement methods.

Reason for the discrepancy in spectral shape, particularly of the calculated losses to the other methods, is found in the evaluation of the input grating coupler loss η_CL,in_. The test structures, where input coupling loss was evaluated for the added component transmission, were located ∼5 mm from the input grating coupler to the SPAD. On this length scale, the accumulated thickness variations from the CMOS substrate and the bottom oxide layer shift the wavelength of maximum transmission under a fixed coupling angle. For shorter wavelengths, the transmission spectrum is more sensitive to this wavelength shift in the interference behavior; thus, the mismatch is more prominent at 633 nm.

The behavior encountered at the target wavelengths over the investigated spectra does not significantly deviate. Again, the measured transmission at the SPAD is significantly decreased in comparison to the calculation at 785 nm. The difference in the calculated values here can equally be attributed to the reflectivity of the TiN layer on the CMOS metal surface.

## Discussion and Outlook

In this work, we have designed, fabricated, and conducted measurements utilizing an interface to efficiently couple light from a photonic waveguide layer to a CMOS detector layer. As simulated, the interface exhibits a coupling efficiency comparable to the state of the art (see Table 1). Others have pursued approaches besides grating couplers assisted by metal reflectors^this work,^ ^17^ such as evanescent couplers,^12,25,26^ slanted metal reflectors,^16^ or grating couplers without auxiliary metal layers.^27,28^ The geometry we presented is significantly more compact than structures implemented for evanescent coupling approaches while maintaining a high coupling efficiency and is on par in size and efficiency with other refractive coupling interfaces.

**Table 1:** Waveguide-to-Photodiode Interface Coupling Efficiencies^a^

Coupling Method	Photodiode	Wavelength (nm)	Coupler Dimensions	Coupling Efficiency (%)	Publication
Evanescent	Si SPAD	850	25 μm	46	^25^
Evanescent	Si p-i-n	850	150 μm	N/A	^26^
Evanescent	Si SPAD	488, 532	100 μm	N/A	^12^
Slanted Reflector	N/A	N/A	10×10 μm^2^	∼50	^16^
Grating	GaAs p-i-n	780/850	15×5 μm^2^	44/56	^27^
Grating	InGaAs SPAD	1550	25×25 μm^2^	50	^28^
Grating + Reflector	Si p-i-n	800–900	>50×50 μm^2^	∼70	^17^
Grating + Reflector	Si SPAD	633	13.6×9.1 μm^2^	58	This work
		785	19.5×7.5 μm^2^	63	

a Simulated coupling efficiencies for suitable interfaces between the two integrated technologies. A selection of parameters from the respective publications is given.

These ultra-compact couplers provide a solution to highly integrated systems, as shown in the demonstration of the designed interface in a post-CMOS integrated photonic circuit. By only using low-temperature (<400 °C), CMOS-compatible processes, which rules out annealing, we fabricated the waveguide layer, as well as the reflector, on top of pre-fabricated 200 mm wafers containing Si SPADs. We implemented two CMP steps to create even SiO_2_ interfaces onto which the waveguide layer and the reflector layer were deposited. Photonic and electronic devices were fully functional after integration, demonstrating the applicability of the developed platform for post-CMOS photonics.^20^ Key metrics of the post-CMOS integrated SPADs, the breakdown voltage *U*_BD_ and dark count rate *f*_DCR_, indicate no impact on the SPADs’ functionality; however, a degradation below the die-to-die variation of these metrics cannot be excluded rigorously due to a missing comparison to a non-integrated state of the SPADs.

We utilized the integrated device to successfully carry out single-photon measurements and used these measurements to evaluate the impact of the metal reflector on transmission. At the target wavelength of 633 nm, the transmission evaluated from the SPAD count rate (Method 2) improved from −34.1 dB to −29.6 dB with the reflector, corresponding to a gain of +4.5 dB. The component-wise transmission estimate (Method 3) improved from −30.8 dB to −23.0 dB, corresponding to a gain of +7.8 dB. These findings are supported by 2D FDTD simulations of the interface, which predict a +5 dB improvement.

At the target wavelength of 785 nm, the transmission evaluated from the SPAD count rate (Method 2) improved from −35.3 dB to −33.2 dB with the reflector, corresponding to a gain of +2.1 dB. The component-wise transmission estimate (Method 3) improved from −23.2 dB to −18.7 dB, corresponding to a gain of +4.5 dB. These findings are supported by 2D FDTD simulations of the interface, which predict an improvement of +6.4 dB.

Overall, the results demonstrate the positive impact of the reflector on coupling efficiency. While the measured, estimated, and simulated values are consistent in the overall trend, the mismatch observed at 785 nm is consistent with the antireflective properties of the TiN-coated CMOS metal surface at this wavelength. These results also identify several opportunities for improving transmission in future device iterations, most notably the modification of the TiN coating on the CMOS metal layer and the optimization of the fiber-to-chip coupling efficiency.

Comparing the different transmission evaluation methods indicates that the experimentally measured (method 2) total transmission is lower than the component-wise estimate (method 3), suggesting the presence of additional loss mechanisms not captured in the current model. Future work will therefore focus on both minimizing the identified loss contributions and improving their characterization. Additional test structures and measurements will be implemented to isolate and quantify individual loss contributions, enabling a more accurate extraction of key device parameters such as the photon detection efficiency of the SPAD and the development of a more complete transmission model. At the same time, transmission will be improved through enhancements in the surface quality of the components involved in the waveguide-to-SPAD interface, removal of the absorptive TiN coating, and implementation of bottom reflectors beneath the input grating couplers to optimize their coupling efficiency. Together, these developments are expected to significantly increase the end-to-end detection efficiency from fiber input to SPAD detection, moving the post-CMOS photonic platform toward performance levels required for practical single-photon sensing applications.

## #

LittleFuse Europe GmbH, 44227 Dortmund, Germany

## Abbreviations

ASIC: application-specific integrated circuits
Al: aluminum
CL: coupling loss
CMOS: complementary metal-oxide-semiconductor
DC: directional coupler
DUT: device under test
DUV: deep ultraviolet
FDE: finite difference eigenmode
FDTD: finite-difference time-domain
FPS: fiber power splitter
IDP: internal detection probability
MMF: multimode fiber
OPM: optical power meter
LOCOS: local oxidation of silicon
PECVD: plasma-enhanced chemical vapor deposition
PIC: photonic integrated circuit
PVD: physical vapor deposition
RIE: reactive ion etching
ROIC: readout integrated circuit
SMF: single-mode fiber
SPAD: single-photon avalanche diode
Si: silicon
SiN: silicon nitride
SiO_2_: silicon dioxide
TEM: transmission electron microscopy
TiN: titanium nitride
WG: waveguide
